# Hunting for Monolayer Oxide Nanosheets and Their Architectures

**DOI:** 10.1038/srep19402

**Published:** 2016-01-25

**Authors:** Hyung-Jun Kim, Minoru Osada, Yasuo Ebina, Wataru Sugimoto, Kazuhito Tsukagoshi, Takayoshi Sasaki

**Affiliations:** 1 International Center for Materials Nanoarchitechtonics (WPI-MANA), National Institute for Materials Science (NIMS), Tsukuba, 305-0044, Japan; 2 Materials and Chemical Engineering, Shinshu University, Ueda, Nagano 386-8567, Japan

## Abstract

In two-dimensional materials, thickness identification with a sufficient characterization range is essential to fundamental study and practical applications. Here, we report a universal optical method for rapid and reliable identification of single- to quindecuple-layers in oxide nanosheets (Ti_0.87_O_2_, Ca_2_Nb_3_O_10_, Ca_2_NaNb_4_O_13_). Because of their wide bandgap nature (*E*_g_ = ∼4 eV) and zero opacity, most oxide nanosheets exhibit a weak white-light contrast (<1.5%), which precludes optical identification. Through a systematic study of the optical reflectivity of Ti_0.87_O_2_ nanosheets on SiO_2_/Si substrates, we show that the use of thinner SiO_2_ (∼100 nm) offers optimum visualization conditions with a contrast of >5%; the contrast is a nonmonotonic function of wavelength and changes its sign at ≈550 nm; the nanosheets are brighter than the substrate at short wavelengths and darker at long ones. Such a nonmonotonic optical response is common to semiconducting oxide nanosheets, including Ca_2_Nb_3_O_10_ and Ca_2_NaNb_4_O_13_. The optical contrast differences between the substrates and nanosheets with different numbers of layers were collected, serving as a standard reference from which the number of layers can be determined by optical microscopy. Our method will facilitate the thickness-dependent study of various oxide nanosheets and their architectures, as well as expedite research toward practical applications.

Two-dimensional (2D) nanosheets with atomic or molecular thickness have been emerging as a new frontier of materials science owing to their unique properties. Inspired by the intriguing properties of graphene, many efforts have been devoted to synthesizing 2D nanosheets of various inorganic materials, including transition-metal dichalcogenides (TMDCs)^1^,^2^, metal oxides^3^,^4^, and hydroxides^3^,^5^, as well as primarily investigating their unique electronic structures and physical properties^1^,^6^,^7^. Among the types of inorganic nanosheets, oxide nanosheets are important and fascinating research targets because of the virtually infinite varieties of layered oxide materials with interesting functional properties, including high-κ ferroelectricity, superconductivity, and magnetism^8^. A variety of oxide nanosheets (such as Ti_1-δ_O_2_, Ti_1-*x*_Co_*x*_O_2_, MnO_2_, and perovskites) have been synthesized by delaminating layered precursors into molecular single sheets *via* a soft-chemical process^3^.

These oxide nanosheets have distinct differences and advantages compared with graphene and other inorganic nanosheets because of their potential uses as insulators, semiconductors, conductors, and even ferromagnets, depending on their composition and structure. Most oxide nanosheets synthesized to date are *d*^0^ transition metal oxides (with Ti^4+^, Nb^5+^, Ta^5+^, W^6+^) with wide-gap semiconducting or insulating nature^7^. Current research on oxide nanosheets has thus focused on their use as semiconducting or dielectric nanoblocks in energy, environmental, and electronic applications. Regarding the fundamental study and practical applications of oxide nanosheets, thickness information and sufficient characterization range is particularly important, but still challenging. Localized techniques such as atomic force microscopy (AFM) or transmission electron microscopy (TEM) are commonly used to measure the thickness of oxide nanosheets^6^,^9^. However, these techniques are time-consuming and unsuitable for rapid measurement over a large area. In the cases of graphene and TMDCs, mono- and few-layer forms are identified by their optical contrasts and Raman signatures^10^,^11^,^12^,^13^. Little is known about these characteristics for oxide nanosheets. Developing a general and effective thickness characterization scheme is highly desirable in the 2D scientific community because it enables the facile fabrication of monolayer devices based on oxide nanosheets.

Here, we report the optical properties of mono- and few-layer titania nanosheets (Ti_1-δ_O_2_) obtained by solution-based exfoliation of a layered titanate^14^,^15^. Because of their zero opacity (the band gap is ∼4 eV), Ti_1-δ_O_2_ nanosheets exhibit a low degree of optical contrast, even if interference enhancement using oxidized Si wafers is employed. We show that the use of thinner SiO_2_ (∼100 nm) offers optimum visualization conditions with a contrast of ∼5% per layer, and this contrast level is sufficient to detect the monolayers under a microscope. To show the versatility of our optical technique, we have extended our research to other oxide nanosheets (Ca_2_Nb_3_O_10_, Ca_2_NaNb_4_O_13_, RuO_2_, MnO_2_) and heterostructures (RuO_2_/Ti_0.87_O_2_, MnO_2_/Ti_0.87_O_2_).

## Results and Discussion

### Optical properties of titania nanosheets

**Figure 1:**
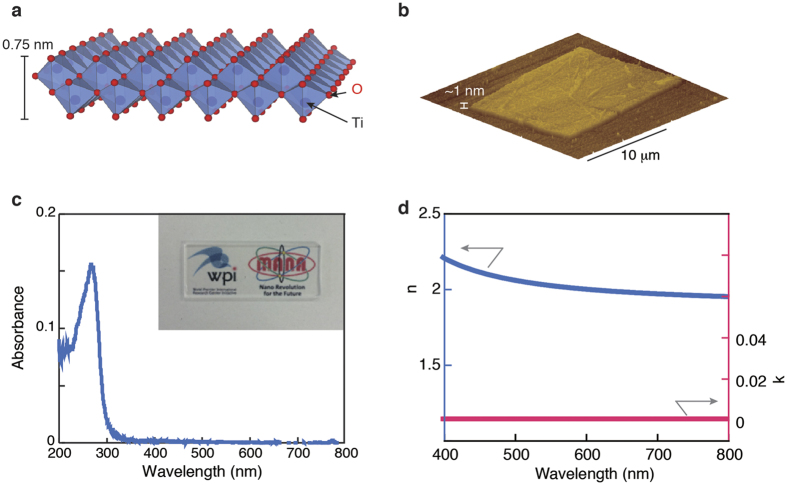
Structure and optical properties of Ti_1−δ_O_2_ nanosheet. (**a**) Structural model of Ti_1-δ_O_2_ nanosheet. Ti atom is coordinated with six oxygen atoms and resulting TiO_6_ octahedra are joined *via* edge-sharing to produce the 2D lattice. Its thickness is ∼0.75 nm, being consisted of two edge-shared TiO_6_ octahedra. (**b**) AFM image of Ti_0.87_O_2_ nanosheet on an oxidized Si substrate. A tapping-mode AFM in vacuum condition was used to evaluate the morphology of the nanosheet. (**c**) Absorbance spectrum for a monolayer film of Ti_0.87_O_2_ nanosheets on a quartz glass substrate. Inset shows a photograph. (**d**) Spectral function of the refractive index (*n*) and extinction coefficient (*k*) of Ti_0.87_O_2_ nanosheet.

Titania nanosheet Ti_1-δ_O_2_ (*δ* ≈ 0.09)^14^,^15^, the initially developed model system of oxide nanosheets, was chosen as the specimen (Fig. 1a,b). Ti_1-δ_O_2_ nanosheets are a new class of nanometer-sized titanium oxide prepared by delaminating a layered titanate into single molecular sheets. Elemental Ti_1-δ_O_2_ nanosheets are characterized by a 2D structure; the thickness is ∼0.75 nm, corresponding to two edge-shared TiO_6_ octahedra^16^. The compositions of the exfoliated 2D nanosheets slightly deviate from the stoichiometry of TiO_2_, with a general formula of Ti_1-δ_□_δ_O_2_^δ−^ (where □ represents vacancies) depending on the starting layered compounds^14^,^15^,^17^. Theoretical and experimental investigations have demonstrated that Ti_0.87_O_2_ nanosheets act as a high-*κ* dielectric, and its multilayer films exhibit a high dielectric constant (*ε*_r_) of ∼125 at thicknesses as low as 10 nm^18^,^19^.

Because of their unique 2D structure and high-*κ* dielectric nature, Ti_0.87_O_2_ nanosheets exhibit some distinctive optical properties in comparison with their bulk counterparts^20^. As shown in Fig. 1c, a sharp absorption peak centered at ∼265 nm was observed for Ti_0.87_O_2_ nanosheets. An analysis of the square root of the absorption edge [*i.e*., (*αhν*)^0.5^] against photon energy (*hν*) provides the information to estimate a band gap energy (*E*_g_) of 3.85 eV, considerably larger than those of bulk anatase, rutile, and the layered parent titanate^20^. We also note that Ti_0.87_O_2_ nanosheets possess a high transmittance (>99%), higher than those of graphene (∼98%)^21^ and MoS_2_ (∼95%)^22^. Fig. 1d depicts the spectral function of the refractive index (*n*) and extinction coefficient (*k*) of a Ti_0.87_O_2_ nanosheet. Ti_0.87_O_2_ nanosheet possessed a higher *n* (>2) and nearly a zero extinction coefficient (*k*), which agree well with the high permittivity value^19^ and previous studies on Ti_0.91_O_2_ case^23^. Due to its zero opacity (the band gap is ∼4 eV), Ti_0.87_O_2_ nanosheet exhibited a low degree of optical contrast, even when employing interference enhancement using oxidized Si wafers. For the standard oxide thickness of 500 nm of SiO_2_, Ti_0.87_O_2_ showed a white-light contrast of <1.5%, which precludes identification using conventional optical microscopy (Supplementary Fig. S1).

### Thickness identification of titania nanosheets by optical microscopy

**Figure 2:**
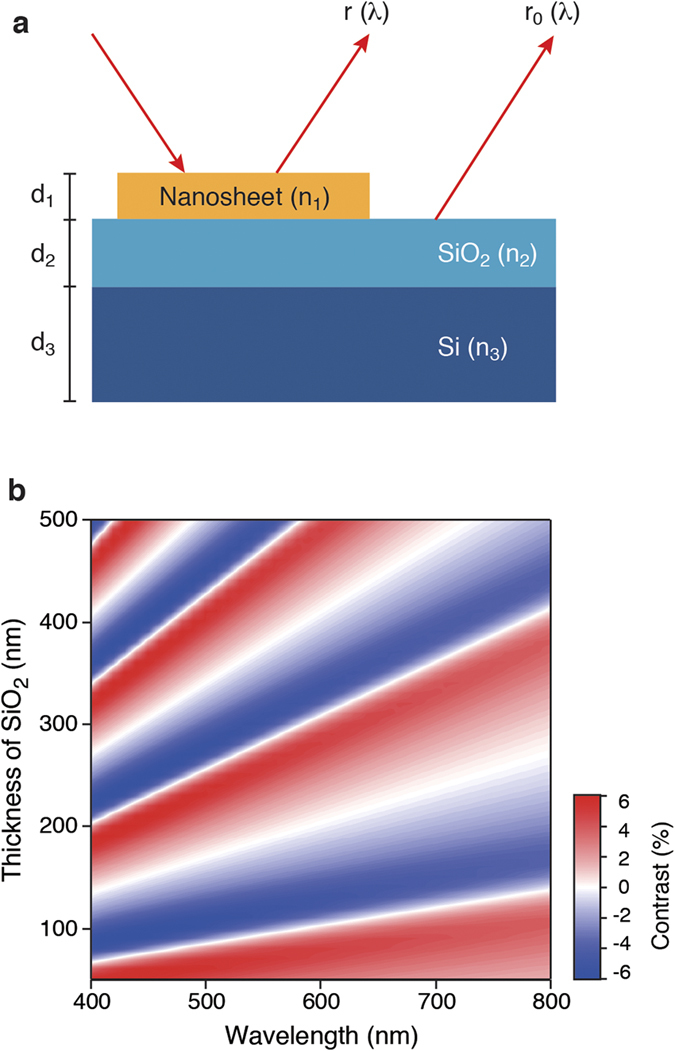
Calculated optical contrast of monolayer Ti_0.87_O_2_ nanosheets. (**a**) Sample geometry used for our analyses. (**b**) Calculated optical contrast of Ti_0.87_O_2_ nanosheets as a function of the wavelength of light and SiO_2_ thickness.

To model the optical contrast of Ti_0.87_O_2_ nanosheets, we employed an analysis based on the Fresnel law, which has been proven to be valid for graphene^10^. In our simulation, we used a matrix formalism of interference in a multilayer system, where the light incident from the air is assumed to be normal to the Ti_0.87_O_2_/SiO_2_/Si structure (Fig. 2a). The reflected light intensity can be expressed as


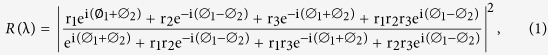


where the subindexes 0, 1, 2, and 3 refer to the medium (air), Ti_0.87_O_2_ nanosheet, SiO_2_, and Si, respectively. *λ* is the wavelength of the inspection light. *r*_1_ = (*n*_0_ − *n*_1_)/(*n*_0_ + *n*_1_),r_2_ = (*n*_1_ − *n*_2_)/(*n*_1_ + *n*_2_), *r*_3_ = (*n*_2_ − *n*_3_)/(*n*_2_ + *n*_3_) are the relative indexes of refraction at the top of the nanosheet surface, the interface between the nanosheet and SiO_2_ and between the SiO_2_ and the Si substrate, respectively. *n*_*i*_ is the refractive index of a given medium. *ϕ*_*i*_ = 2*πn*_*i*_*d*_*i*_/*λ* is the phase shift due to the light passing through a given medium, where *d*_*i*_ is the thickness of the medium *i*. The optical contrast of the system can be defined as


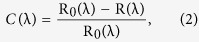


where R_*0*_ and R and are the intensities of reflected light from the SiO_2_/Si substrate and the nanosheets, respectively. We used spectroscopic ellipsometry data for 5-layer films of Ti_0.87_O_2_ nanosheets and found k ∼ 0 and n ∼ 2.1. Assuming that the optical properties of monolayers change little with respect to 5-layer films, we obtain the dependences shown in Fig. 2b. The developed theory allows us to predict the SiO_2_ thickness at which the optical contrast for monolayer Ti_0.87_O_2_ nanosheets would be maximal; here, a contrast peak is predicted at a thickness of 100 nm. In this case, the contrast remains relatively strong (>5%) over a wide range of visible wavelengths (400–550 nm). Moreover, the contrast changes from negative to positive when crossing from the blue-light to the red-light region of the spectrum, going through zero in the green-light region.

**Figure 3:**
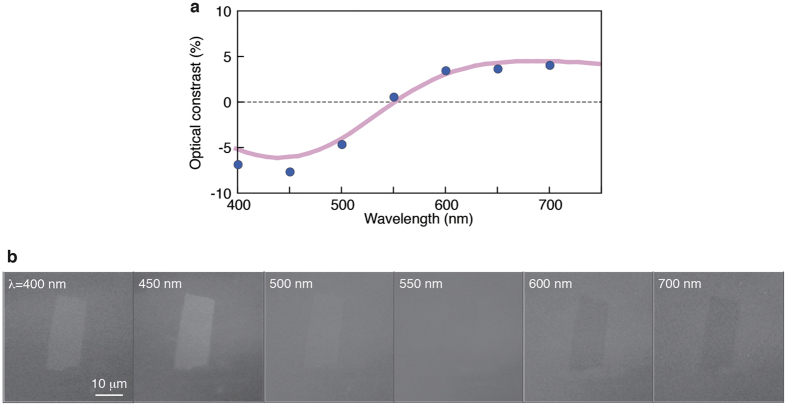
Optical identification of Ti_0.87_O_2_ nanosheets. (**a**) Optical contrast of monolayer Ti_0.87_O_2_ nanosheets on a 100 nm SiO_2_/Si substrate (blue circle: experimental value, pink line: theoretical prediction). (**b**) Optical images of Ti_0.87_O_2_ nanosheets were taken at selected wavelengths centered at 400, 450, 500, 550, 600, and 700 nm.

This prediction has been confirmed experimentally by imaging monolayer Ti_0.87_O_2_ nanosheets on a 100 nm SiO_2_/Si substrate (Fig. 3a).We also investigated the optical contrast of monolayer Ti_0.87_O_2_ nanosheets on SiO_2_/Si substrates with different SiO_2_ thicknesses (90 and 285 nm) (Supplementary Fig. S2). Several observations can be made from these data. The contrast for Ti_0.87_O_2_ nanosheets can be both negative and positive depending on the wavelength, with a zero crossing in between. A contrast of zero means that the nanosheet is invisible at that wavelength, *i.e*., it has the same reflectivity as the substrate. The negative contrast was stronger, with a peak observed at ∼450 nm, while the positive contrast appeared at >550 nm. On the bottom panels (Fig. 3b), we show optical images of Ti_0.87_O_2_ nanosheets taken at selected wavelengths centered at 400, 450, 500, 550, 600, and 700 nm. To acquire the image, we have taken optical micrographs using illumination through narrow bandpass filters (with a width of ±5 nm). Clearly, Ti_0.87_O_2_ nanosheets showed greater reflectivity than the substrate at 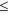
450 nm (*i.e*., negative contrast) and lesser reflectivity than the substrate at 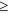
600 nm (*i.e.*, positive contrast). The 550-nm image corresponds to the wavelength showing zero contrast. Comparing the observed and calculated values, the theory accurately reproduces the observed contrast (Fig. 3a), including its reversal at 550 nm. We note that the optical contrast at 450 nm reaches ∼5% per layer, and this contrast level is comparable to those of graphene and TMDCs^10^,^11^,^12^,^13^.

**Figure 4:**
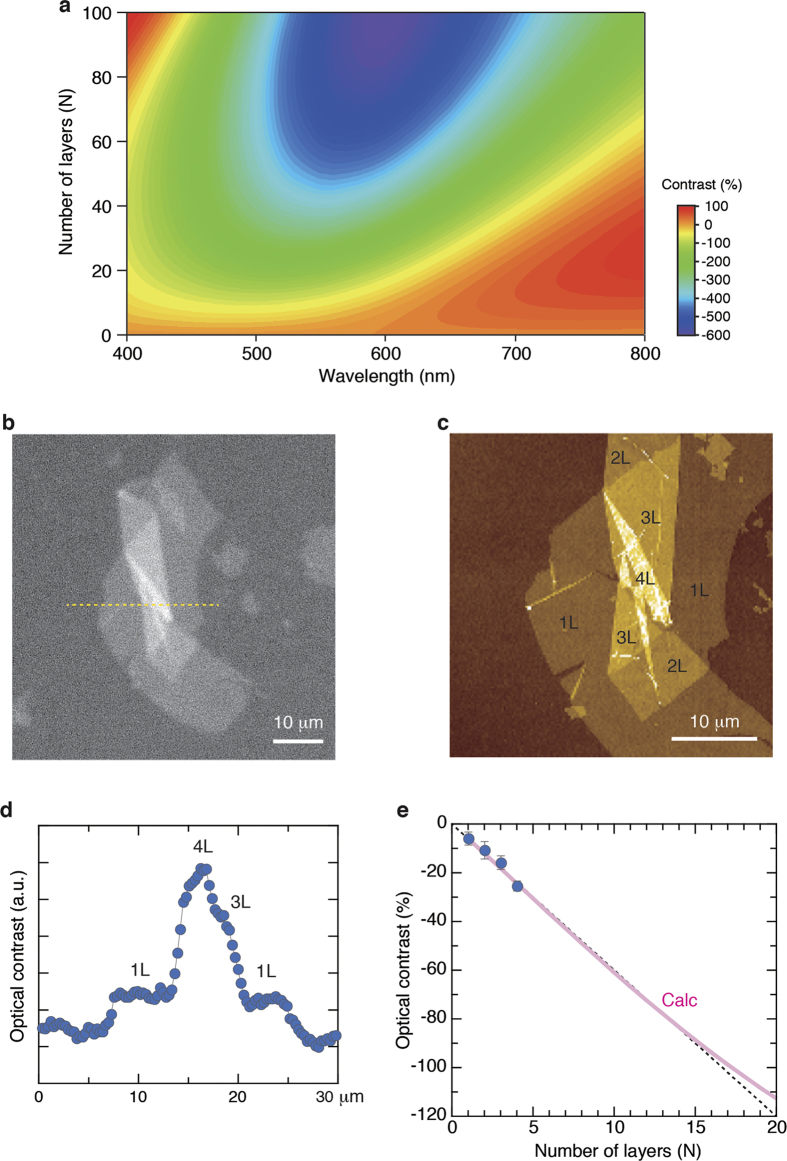
Thickness identification of Ti_0.87_O_2_ nanosheets by optical microscopy. (**a**) Calculated optical contrast of Ti_0.87_O_2_ nanosheets as a function of the wavelength of light and number of layers (*N*). (**b**) Optical image for Ti_0.87_O_2_ nanosheets on a 100 nm SiO_2_/Si substrate. Image was taken with the 450-nm light, which is near the negative peak. (**c**) AFM image taken from the same film as (**b**). This image clearly revealed different thicknesses ranging from 1 to 4 layers. (**d**) A line profile at the selected area in (b). The trace shows step-like changes in the contrast for 1, 2, 3, and 4 layers. (**e**) Comparison between the observed (blue circle) and theoretically predicted (pink line) optical contrast for Ti_0.87_O_2_ nanosheets. A trend of a linear increase of the optical contrast is persistent up to *N* ≈ 15.

The optical contrast also depends on the number of layers (*N*) of Ti_0.87_O_2_ nanosheets. From a similar analysis as the one presented in Fig. 2, we investigated the layer dependence of the optical contrast for Ti_0.87_O_2_ nanosheets on a 100 nm SiO_2_/Si substrate (Fig. 4a, Supplementary Fig. S3). This calculation suggests that the use of the 450-nm light is suitable for monitoring the layer dependence in Ti_0.87_O_2_ nanosheets. In Fig. 4b, we show the optical image taken with the 450-nm light, which is near the negative peak. The AFM image (Fig. 4c) revealed different thicknesses ranging from 1 to 4 layers. From the line profiles at the selected area (Fig. 4d), the optical contrast increased in integer steps (by a factor of *N* for *N* layers of Ti_0.87_O_2_ nanosheets), a trend consistent with the theoretical prediction (Fig. 4e). In this study, we evaluated thicknesses up to 4 layers. A trend of a linear increase of the optical contrast is persistent up to *N* ≈ 15. These results imply that the contrast observation at a single wavelength (450 nm) can be used for rapid and reliable characterization of the thickness of Ti_0.87_O_2_ nanosheets. We also note that the typical acquisition time for an optical image is ∼500 ms, much shorter than that of AFM, which is on the order of 10 min.

### Optical identification in 2D oxide nanosheets and their architectures

**Figure 5:**
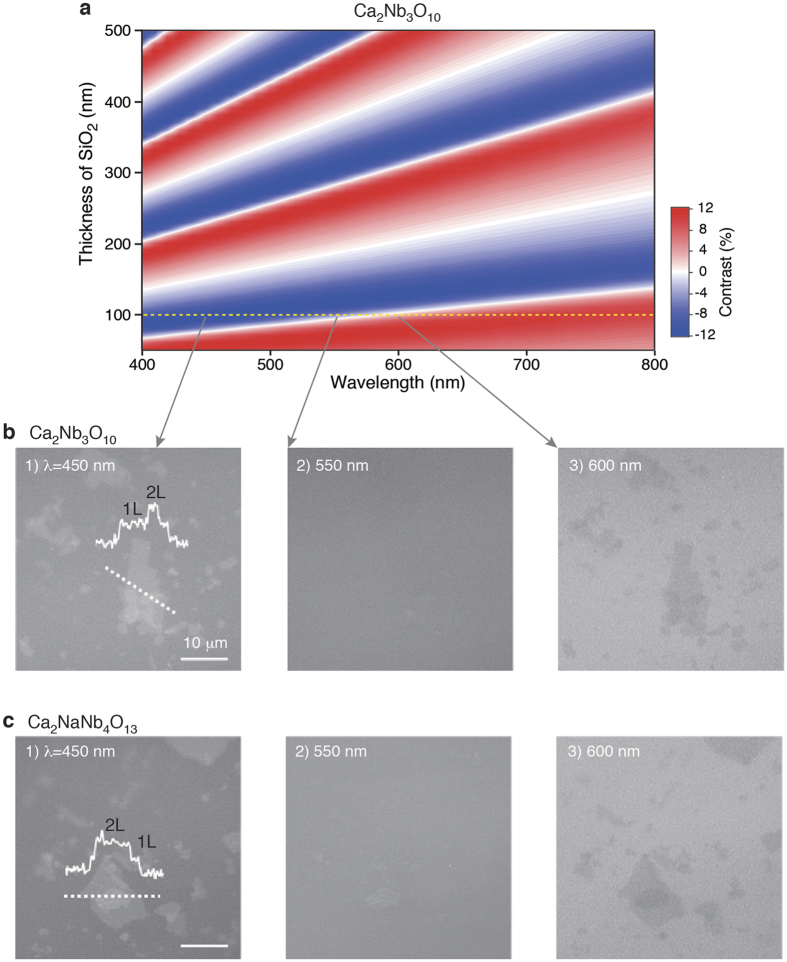
Optical identification of perovskite nanosheets. (**a**) Calculated optical contrast of Ca_2_Nb_3_O_10_ nanosheets as a function of the wavelength of light and SiO_2_ thickness. (**b,c**) Optical images for Ca_2_Nb_3_O_10_ and Ca_2_NaNb_4_O_13_ nanosheets on a 100 nm SiO_2_/Si substrate. Images were taken at selected wavelengths centered at (1) 450 nm, (2) 550 nm, and (3) 600 nm. Insets in (b-1) and (c-1) present the line profiles of multilayer parts.

Through a systematic study of the optical reflectivity of Ti_0.87_O_2_ nanosheets on SiO_2_/Si substrates, we show that the use of thinner SiO_2_ (∼100 nm) offers optimum visualization, with an optical contrast of >5%. A particular feature of the Ti_0.87_O_2_ nanosheets is that the contrast is a nonmonotonic function of *λ* and changes its sign at ≈550 nm; the nanosheets are brighter than the substrate at short wavelengths and darker at long ones. These features are different from those of graphene and TMDCs, in which the contrast is either positive or negative^10^,^11^,^12^,^13^. We note that the nonmonotonic optical response is common to 2D nanosheets with wide-gap semiconducting or insulating nature, including *h*-BN, Ca_2_Nb_3_O_10_, and Ca_2_NaNb_4_O_13_. A particular feature of these materials is zero opacity, causing a nearly zero extinction coefficient (*k*). Actually, perovskite nanosheets (Ca_2_Nb_3_O_10_, Ca_2_NaNb_4_O_13_) with higher *ε*_r_ (>200)^7^ also showed a nonmonotonic response of the optical contrast; the nanosheets were brighter than the substrate at short wavelengths and darker at long ones (Fig. 5). The optical contrast also depends on the number of layers (*N*) of perovskite nanosheets, a situation being similar to that of Ti_0.87_O_2_ nanosheets (Fig. 4, Supplementary Fig. S5). The calculation on the thickness dependence suggests that the use of the 450-nm light is suitable for monitoring the layer dependence in Ca_2_Nb_3_O_10_ nanosheets; a trend of a linear increase of the optical contrast is persistent up to *N* ≈ 12. Despite their high-*κ* nature (*i.e.*, higher *n*), these nanosheets afforded higher optical contrast compared to Ti_0.87_O_2_ nanosheets, reaching ∼10%. This is probably due to both the reduced band gap (*E*_g_ ∼ 3.4 eV) and slightly larger thicknesses (1.8 nm for Ca_2_Nb_3_O_10_, 2.2 nm for Ca_2_NaNb_4_O_13_). In this context, *h*-BN nanosheets possess a large band gap (∼5 eV), causing a rather low contrast (<2.5%)^24^,^25^; it is still much harder to detect *h*-BN than graphene and oxide nanosheets.

**Figure 6:**
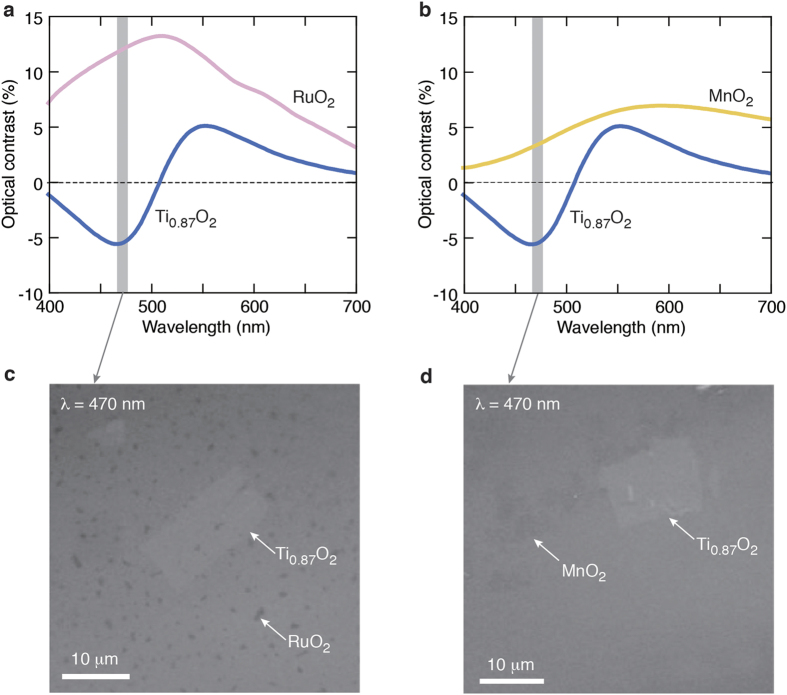
Optical identification of nanosheet heterostructures [RuO_2_/Ti_0.87_O_2_ and MnO_2_/Ti_0.87_O_2_]. (**a,b**) Calculated optical contrast of monolayer RuO_2_/Ti_0.87_O_2_ and MnO_2_/Ti_0.87_O_2_ on a 90 nm SiO_2_/Si substrate. The use of thinner SiO_2_ (∼90 nm) offers optimum visualization; here, RuO_2_ and MnO_2_ cause the positive contrast, while Ti_0.87_O_2_ the negative contrast (Supplementary Fig. S5, S6). RuO_2_ and MnO_2_ are either metallic or semi-metallic, causing a strong negative contrast with respect to Ti_0.87_O_2_ and SiO_2_/Si substrate. (**c,d**) Optical images for RuO_2_/Ti_0.87_O_2_ and MnO_2_/Ti_0.87_O_2_ on a 90 nm SiO_2_/Si substrate. Images were taken with the 470-nm light, which is near the maximal contrast for Ti_0.87_O_2_ nanosheets.

Our optical identification method will facilitate the thickness-dependent study of various 2D oxide nanosheets and their architectures. An attractive aspect is that oxide nanosheets can be organized into various nanoarchitectures by applying a solution-based layer-by-layer assembly method^23^. Sophisticated functionalities or nanodevices can be designed through the selection of nanosheets and combining materials^6^,^7^,^26^,^27^,^28^,^29^,^30^,^31^. Fig. 6 shows a practical application of this imaging method; we extend our research to heterostructures such as RuO_2_/Ti_0.87_O_2_ and MnO_2_/Ti_0.87_O_2_. These structures are basic components of nanocapacitors^30^ and photo conversion devices^27^. In this study, we prepared hetero-assembled structures on SiO_2_/Si substrates by a drop-casting method. Through a systematic study of the optical reflectivity of RuO_2_/Ti_0.87_O_2_ and MnO_2_/Ti_0.87_O_2_ on SiO_2_/Si substrates, we show that the use of thinner SiO_2_ (∼90 nm) and shorter wavelength light (λ = 470 nm) offers optimum visualization; RuO_2_ and MnO_2_ cause the positive contrast at ∼470 nm, while Ti_0.87_O_2_ the negative contrast (Supplementary Fig. S5, S6). RuO_2_ and MnO_2_ are either metallic or semi-metallic, causing a strong positive contrast (5 ∼ 10%) with respect to Ti_0.87_O_2_ and SiO_2_/Si substrate. We also emphasize that our technique offers rapid thickness identification with a sufficient characterization range (even on the sub mm scale). These results imply the versatility of our optical technique for the rapid and reliable characterization of various 2D oxide nanosheets and their architectures.

## Methods

### 2D Oxide Nanosheets

A colloidal suspension of Ti_0.87_O_2_ nanosheets with a lateral dimension of 5–10 *μ*m was prepared by delaminating a layered titanate (K_0.8_Ti_1.73_Li_0.27_O_4_) according to previously reported procedures^14^,^15^,^17^. Ti_0.87_O_2_ nanosheets were deposited on SiO_2_/Si substrates (SiO_2_ thicknesses: 90, 100, 285, and 300 nm) by a modified Langmuir-Blodgett (LB) technique^32^ (Supplementary Fig. S7). In usual LB experiments, densely packed monolayer films were obtained with an optimized surface pressure (∼15 mN/m). In this study, films having dispersed nanosheets were obtained by controlling the surface pressure (∼3 mN/m). The as-fabricated films were irradiated by UV/white light from a Xe lamp (4 mW/cm^2^) for 48 h to decompose the tetrabutylammonium ions used in the exfoliation process. Repeated LB deposition yielded multilayer structures. The main data were obtained from Ti_0.87_O_2_ nanosheets with different thicknesses ranging from 1 to 4 layers. Complementary data were obtained from perovskite nanosheets (Ca_2_Nb_3_O_10_, Ca_2_NaNb_4_O_13_), RuO_2_, MnO_2_, and heterostructures (RuO_2_/Ti_0.87_O_2_, MnO_2_/Ti_0.87_O_2_). The films with dispersed nanosheets were prepared by a drop-casting method. The synthesis and characterization of these nanosheets were described elsewhere^30^,^33^,^34^,^35^.

### Optical Microscopy of 2D Nanosheets

Optical images of 2D oxide nanosheets were obtained by a bright-field optical microscope (Olympus, BX51 with a 100×, 0.9 NA objective lens). Monochromatic images were acquired using illumination through narrow bandpass filters (with a width of ±5 nm). A CCD camera head (Nikon, DS-1) with a digital camera control unit was used to capture color optical images of the 2D nanosheets at the resolution of 1280 × 960 pixels. The typical acquisition time was ∼500 ms; in low-contrast cases, acquisition times were varied between 100 ms and 30 s to avoid overexposure. The color optical images were processed by Image-J software (version 1.48, National Institutes of Health, USA). For color images (RGB images), the contrast value of each pixel (*C*_V_) was calculated using the following equation:


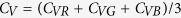


where *C*_VR_, *C*_VG_, and *C*_VB_ are the R, G, and B values per pixel, respectively (0–255, corresponding to darkest to brightest). The contrast levels of the R, G, and B channels were extracted and converted to a gray-scale image, where 0 is black and 255 is white.

To model the optical contrast of oxide nanosheets, we employed an analysis based on the Fresnel law, which has been proven to be valid for graphene^10^. In our simulation, we used a matrix formalism of interference in a multilayer system (Fig. 2a). We used spectroscopic ellipsometry data for 5-layer films of oxide nanosheets. Ellipsometric measurements were performed by a spectroscopic ellipsometer (J.A. Woollam Japan, M-2000).

### Thickness Measurements by AFM

An AFM (SII Nanotech, E-Sweep) was used to confirm the number of layers of 2D oxide nanosheets by measuring the film thickness with tapping mode in air.

## Additional Information

**How to cite this article**: Kim, H.-J. *et al.* Hunting for Monolayer Oxide Nanosheets and Their Architectures. *Sci. Rep.* **6**, 19402; doi: 10.1038/srep19402 (2016).

## Supplementary Material

Supplementary Information
