# Supplementary material for: Hunting for Monolayer Oxide Nanosheets and Their Architectures
Source: Sci Rep. 2016 Jan 25;6:19402. doi: 10.1038/srep19402 (PMC4726153; doi:10.1038/srep19402)
Supplement: Supplementary Information [file srep19402-s1.pdf]

Supplementary Information

Hunting for Monolayer Oxide Nanosheets and Their Architectures

Hyung-Jun Kim[1], Minoru Osada[1*], Yasuo Ebina[1], Wataru Sugimoto[2], Kazuhito Tsukagoshi[1] & Takayoshi Sasaki[1]

[1]*International Center for Materials Nanoarchitechtonics (WPI-MANA), National Institute for Materials Science (NIMS), Tsukuba, 305-0044, Japan*

[2]*Materials and Chemical Engineering, Shinshu University, Ueda, Nagano 386-8567, Japan*

**osada.minoru@nims.go.jp*

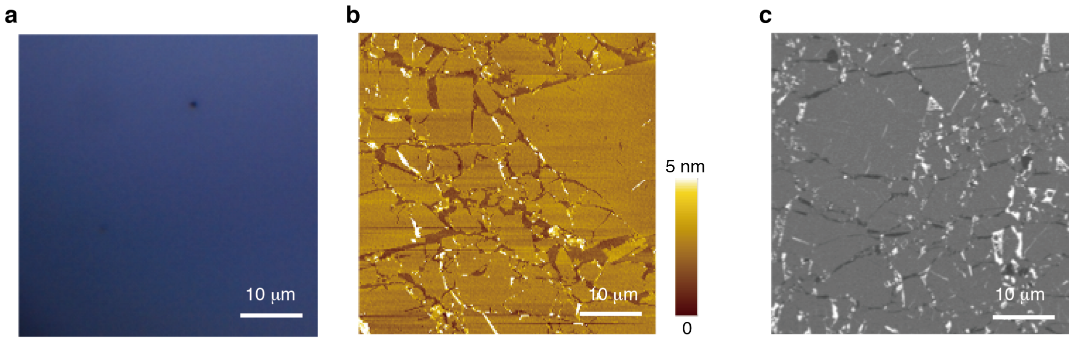

Figure S1. Microscopic observation of monolayer $Ti_{0.87}O_2$ nanosheets on an oxidized Si wafer. (a) Bright-field optical microscopy, (b) AFM, and (c) SEM.

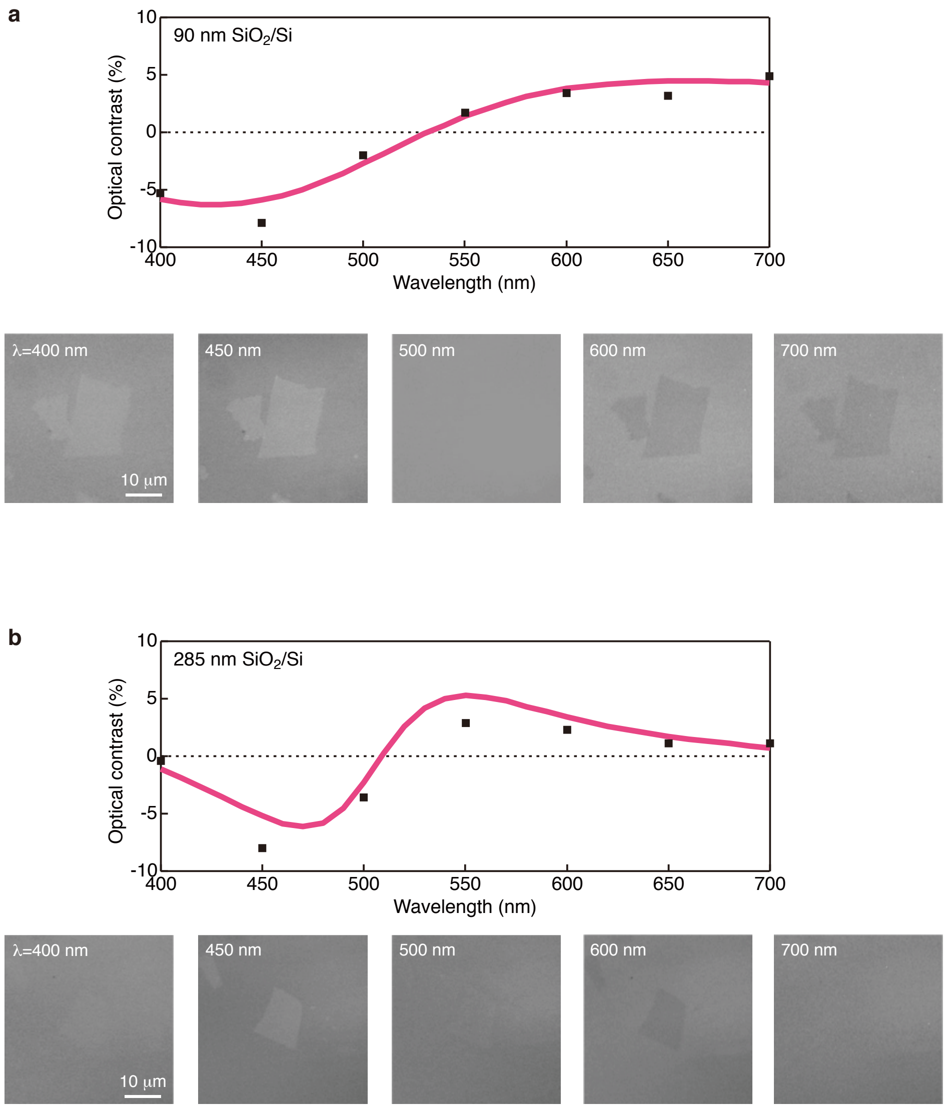

Figure S2. Optical contrast of monolayer $Ti_{0.87}O_2$ nanosheets on SiO_2/Si substrates with different SiO_2 thicknesses [(a) 90 and (b) 285 nm]. Filled squires denote experimental values, while a pink line represents theoretical prediction. On the bottom panels, we show optical images of $Ti_{0.87}O_2$ nanosheets taken at selected wavelengths centered at 400, 450, 500, 600, and 700 nm.

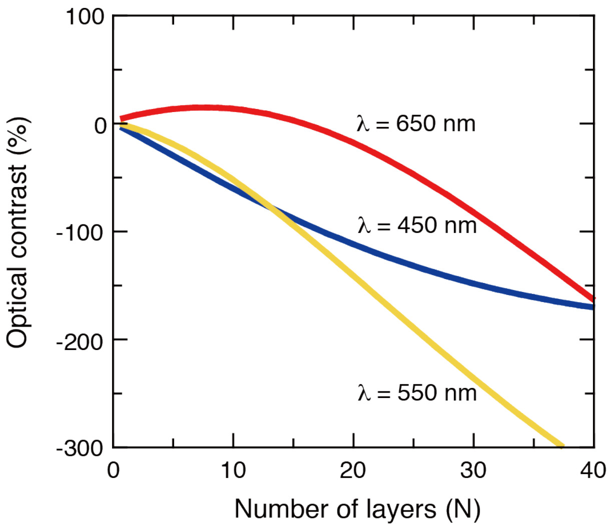

Figure S3. Calculated optical contrast of $Ti_{0.87}O_2$ nanosheets as a function of the number of layers (*N*). We monitored the optical contrast at selected wavelengths at 450, 550, and 650 nm. For the longer wavelengths (550 and 650 nm), the contrast is a nonmonotonic function of *N*. For the 450 nm, on the other hand, a linear increase of the optical contrast is persistent up to $N \approx 15$. These results imply that the use of the 450-nm light is suitable for monitoring the layer dependence in $Ti_{0.87}O_2$ nanosheets.

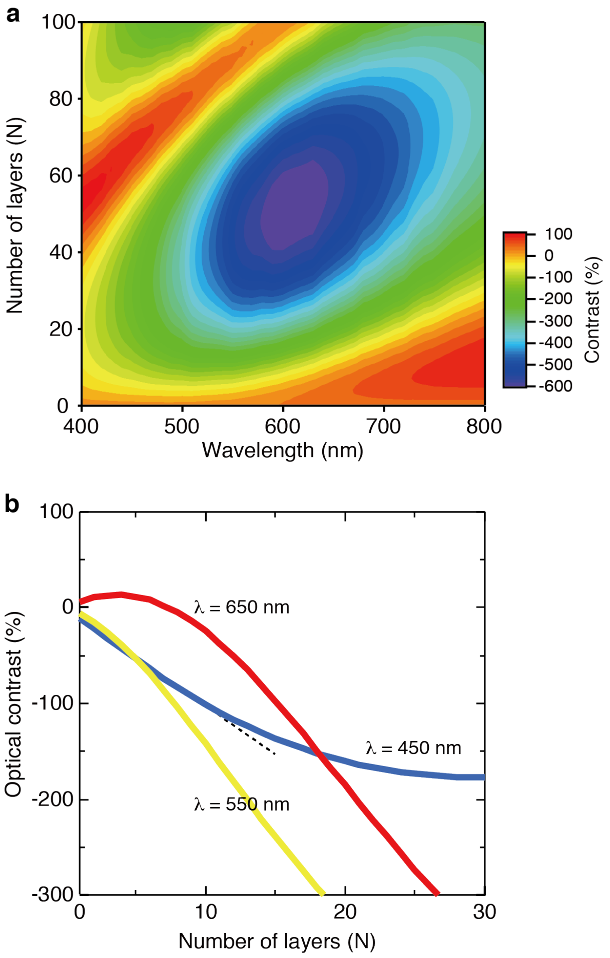

Figure S4. (a) Calculated optical contrast of $Ca_2Nb_3O_{10}$ nanosheets as a function of the wavelength of light and number of layers (*N*). (b) The thickness dependence of the optical contrast at selected wavelengths at 450, 550, and 650 nm. For the longer wavelengths (550 and 650 nm), the contrast is a nonmonotonic function of *N*. For the 450 nm, on the other hand, a linear increase of the optical contrast is persistent up to $N \approx 12$. These results imply that the use of the 450-nm light is suitable for monitoring the layer dependence in $Ca_2Nb_3O_{10}$ nanosheets.

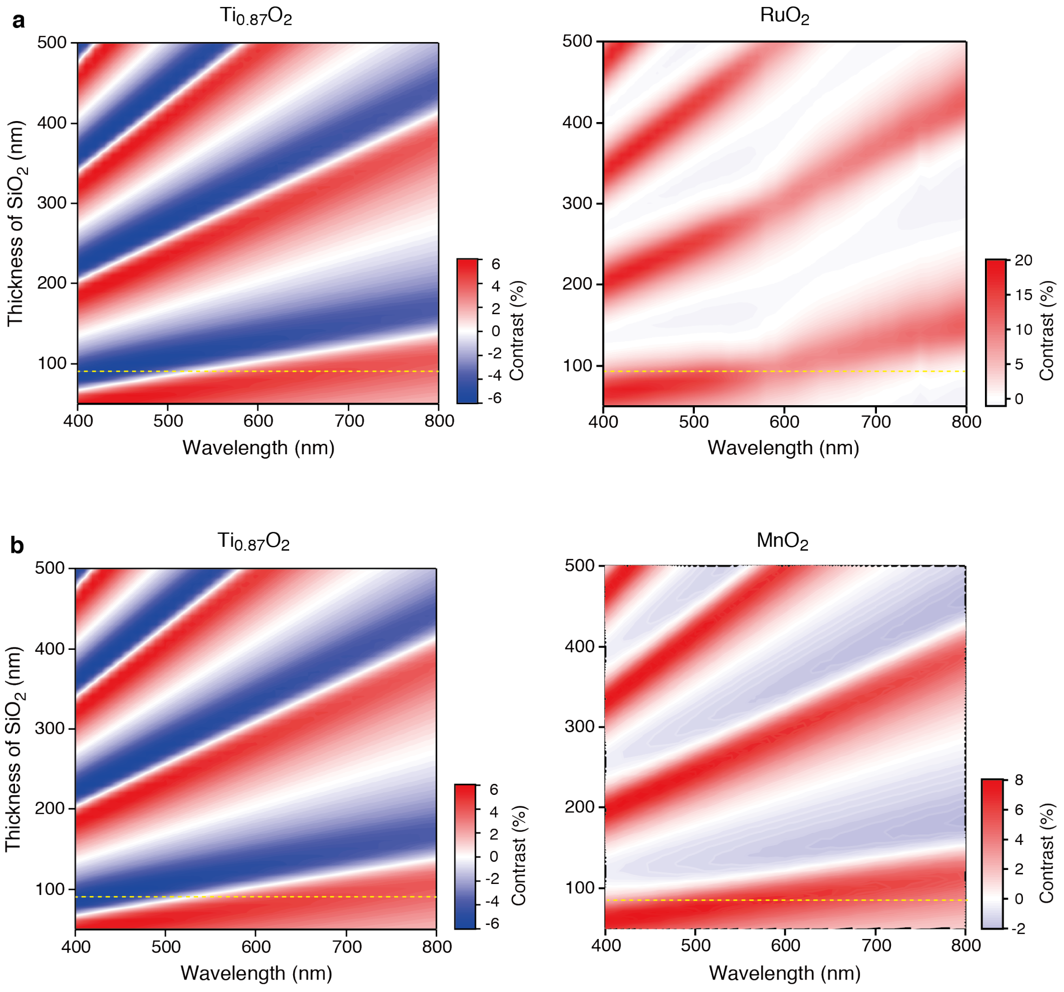

Figure S5. Calculated optical contrasts of (a) $RuO_2/Ti_{0.87}O_2$ and (b) $MnO_2/Ti_{0.87}O_2$ on different SiO_2/Si substrates. The use of thinner SiO_2 (~90 nm) and shorter wavelength light (λ = 470 nm) offers optimum visualization; here RuO_2 and MnO_2 cause the positive contrast, while $Ti_{0.87}O_2$ the negative contrast.

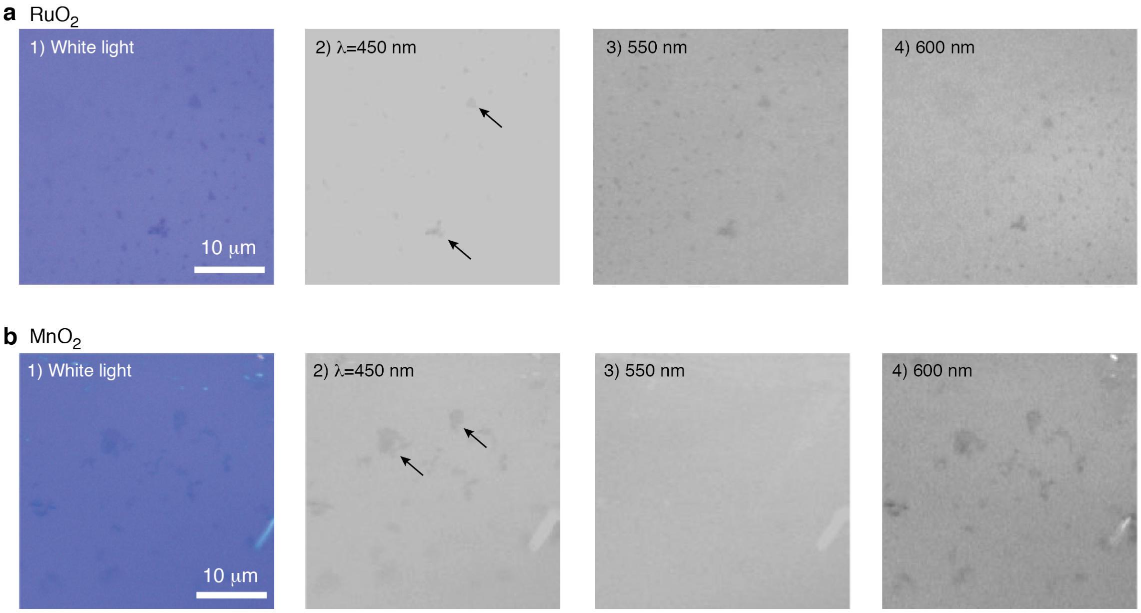

Figure S6. Optical contrast of RuO_2 and MnO_2 nanosheets on a 90-nm SiO_2/Si substrate. Images were taken with (1) a white light and monochromatic light at selected wavelengths at (2) 450, (3) 550, and (4) 600 nm.

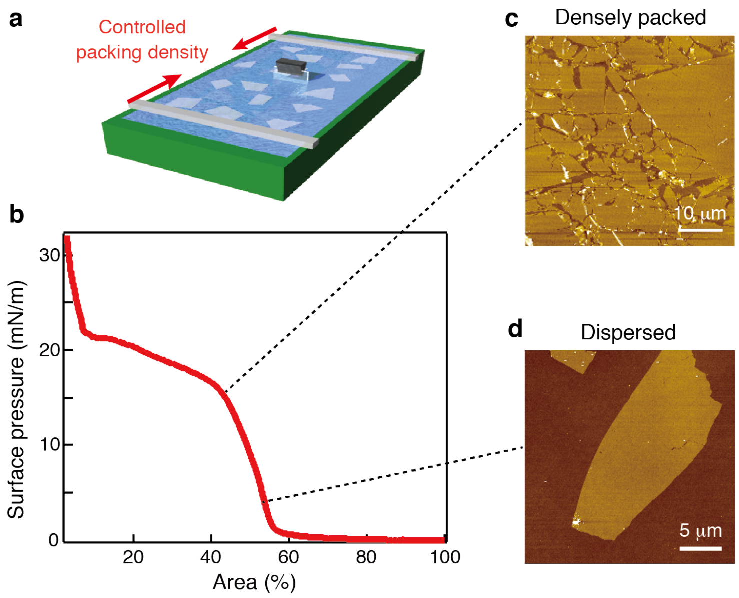

Figure S7. (a) Schematic illustration for a modified LB process. (b) Pressure-area (π-A) isotherm for nanosheet suspension ($Ti_{0.87}O_2$) at 25 °C. For the horizontal axis, the percentage of surface area relative to the area before compression is used. (c, d) AFM images of densely packed and dispersed LB films of $Ti_{0.87}O_2$ nanosheets. In usual LB experiments, densely packed monolayer films (c) were obtained with an optimized surface pressure (~15 mN/m). In this study, films having dispersed nanosheets (d) were prepared by controlling the surface pressure (~3 mN/m).
